# Concurrent Associations of Gaming Time and Problematic Gaming with Depressive Symptoms in Children and Adolescents: A Three-Wave Cohort Study with Complementary Machine Learning Classification

**DOI:** 10.3390/healthcare14193315

**Published:** 2026-10-05

**Authors:** Yeseo Lee, Jun Seok Her, Hyo Jung Kim

**Affiliations:** 1 Department of Artificial Intelligence, College of Engineering, The Catholic University of Korea, Bucheon 14662, Gyeonggi-do, Republic of Korea; cuk24.yeseo@gmail.com; 2 Department of Biomedical Software Engineering, College of Biomedical Sciences and Bioengineering, The Catholic University of Korea, Bucheon 14662, Gyeonggi-do, Republic of Korea; jason5125@catholic.ac.kr

**Keywords:** depression, video games, problematic gaming, adolescent, cohort studies, machine learning

## Abstract

**Highlights:**

**What are the main findings?**

- Problematic gaming symptoms (IGUESS) were consistently associated with concurrent depressive symptoms across three assessment waves at both the between-person and within-person levels, whereas associations with gaming time were model-dependent.
- Machine learning models using gaming-related indicators achieved ROC-AUCs of 0.85–0.88 for concurrent classification of elevated depressive symptoms, although precision was modest.

**What are the implications of the main findings?**

- Assessment of depressive symptoms in relation to gaming may benefit from considering problematic gaming symptoms alongside gaming time.
- Gaming-related indicators may provide complementary, rather than standalone, information for assessing depressive symptoms in children and adolescents.

**Abstract:**

**Background/Objectives**: Digital media use is integral to the daily lives of children and adolescents, intensifying concerns regarding potential associations with mental health. However, much of the literature linking gaming and depression is based on cross-sectional designs and does not adequately distinguish within-person change from between-person differences. This study examined concurrent associations between gaming use patterns, problematic gaming behavior, and depressive symptoms across three repeated assessment waves and, as a complementary analysis, evaluated the concurrent classification performance of gaming-related indicators for elevated depressive symptoms. **Methods**: Three-wave data collected at baseline, 12 months, and 24 months were obtained from the Internet, Game, and Smartphone User Cohort established by the National Center for Mental Health. Depressive symptoms were assessed using the Children’s Depression Inventory (CDI), and problematic gaming behavior was assessed using the Internet Game Use-Elicited Symptom Screen (IGUESS). Linear mixed-effects models, including within–between decomposition and change-score models, were applied. Logistic Regression, Random Forest, and XGBoost were used to classify concurrent elevated depressive symptoms (CDI ≥ 22). **Results**: The primary mixed-effects analyses included 2194 of 2294 enrolled participants. Across models, higher IGUESS scores were consistently associated with greater concurrent depressive symptoms, whereas gaming time showed weak or inconsistent associations. Machine learning models yielded ROC-AUC values of 0.85–0.88; however, precision and F1-scores were modest, indicating a substantial false-positive burden in this low-prevalence setting. **Conclusions**: Overall, problematic gaming behavior, rather than gaming time alone, may be more closely associated with depressive symptoms in children and adolescents.

## 1. Introduction

Digital media use, including internet and smartphone use, has become integral to the daily lives of children and adolescents, intensifying concerns regarding its potential associations with mental health [1]. Gaming, in particular, serves not only as a leisure activity but also as a means of social interaction and stress relief. However, excessive or problematic gaming has emerged as an important public health concern because of its associations with mental health problems, including depression and anxiety [2,3,4]. Clarifying the relationship between gaming behavior and depression is therefore important for informing prevention and intervention strategies in child and adolescent mental health.

Previous studies have generally reported significant associations between gaming behavior and depressive symptoms [5,6,7]. More recent research has further suggested that psychosocial and contextual factors, including gaming motives, family characteristics, parental influences, and psychological distress, may contribute to the relationship between gaming behavior and mental health [6,8,9,10]. However, much of the existing evidence is based on cross-sectional, single-time-point analyses [11,12,13]. Although studies examining gaming and depression across repeated assessments are increasing [9,13], most have considered either gaming time or problematic gaming symptoms in isolation. Studies simultaneously evaluating both across repeated assessment waves, thereby distinguishing associations related to gaming quantity from those related to symptomatic gaming behavior, remain limited [14]. Moreover, conventional analyses that do not separate between-person differences from within-person changes constrain interpretation [15,16], leaving unclear whether increases in problematic gaming within an individual are accompanied by increases in depressive symptoms. Beyond explanatory modeling, predictive and discriminative approaches have increasingly been applied in psychology and mental health [17,18,19], including approaches that quantify how well gaming-related indicators discriminate concurrent elevated depressive symptoms.

Accordingly, this study investigated concurrent associations among gaming use patterns, problematic gaming behavior measured using the Internet Game Use-Elicited Symptom Screen (IGUESS), and depressive symptoms measured using the Children’s Depression Inventory (CDI), repeatedly assessed across three annual waves in cohort data established by the National Center for Mental Health while distinguishing within-person variation from between-person differences. Linear mixed-effects models were used to account for repeated measurements over time and between-person variability and to characterize concurrent associations between gaming behavior and depressive symptoms across assessment waves. As a complementary analysis, machine learning–based models were applied to evaluate the concurrent classification performance of gaming-related indicators for elevated depressive symptoms in children and adolescents. Together, these approaches were intended to provide complementary perspectives from repeated-measures association analyses and concurrent classification analyses.

## 2. Materials and Methods

### 2.1. Data Source

This observational study used repeated-measures data from the “Cohort Study of Internet, Game, and Smartphone Users,” established and managed by the National Center for Mental Health. At baseline, the cohort included children in the third and fourth grades of elementary school, adolescents in the first year of middle school, and their parents, with annual follow-up thereafter. Baseline assessments were conducted in 2015–2016, followed by 12-month assessments in 2016–2017 and 24-month assessments in 2017–2018. The cohort included 2294 participants contributing 6583 observations across the baseline, 12-month, and 24-month assessments. Details of analytic sample construction, including the handling of invalid responses and missing data, are provided in Section 2.3 (Data Preprocessing).

### 2.2. Measures

#### 2.2.1. Outcome: Depressive Symptoms (CDI)

Depressive symptoms were assessed using the CDI, a 27-item self-report questionnaire with total scores ranging from 0 to 54; higher scores indicate greater depressive symptom severity. CDI total scores at each assessment wave were used as the outcome variable [20].

#### 2.2.2. Main Exposures

##### Gaming Use Patterns (Weekly Gaming Time)

At each assessment, total weekly gaming time was calculated as five times the average weekday gaming time plus two times the average weekend or public-holiday gaming time. Responses recorded separately in hours and minutes were converted to minutes before calculation. Weekly gaming time was restricted to the theoretically possible range of 0–10,080 min and log-transformed as log(1 + weekly minutes) to reduce right skewness. Detailed equations and source-variable definitions are provided in Supplementary Materials S1.

##### Problematic Gaming Behavior (Internet Game Use-Elicited Symptom Screen, IGUESS)

Problematic gaming behavior was assessed using the IGUESS, a 9-item questionnaire based on the DSM-5 criteria for Internet Gaming Disorder. Total scores ranged from 0 to 27, with higher scores indicating greater severity of problematic gaming behavior. IGUESS total scores at each assessment wave were used as the primary exposure variable [21].

#### 2.2.3. Covariates (Baseline)

Baseline covariates included sex (SEX), parental education level (ELPR), parental occupation (JOBPR), parental marital status (MAST), parental living standard (LIFELV), and psychosocial factors comprising the School Bullying Scale (SCHOST), Self-Esteem Scale (SES), Social Support Appraisals Scale (SSAS), and Patient Health Questionnaire-9 for parental depressive symptoms (PHQ9).

#### 2.2.4. Additional Baseline Factors (Extensions)

Additional baseline factors included content diversity (GMEP), parental intervention (ONGR), age at first gaming use, and nighttime gaming use. These variables were derived from baseline questionnaire responses according to predefined coding rules (Supplementary Materials S1) and included in additional moderation analyses. Values were coded as missing when all corresponding items were unavailable.

### 2.3. Data Preprocessing

#### 2.3.1. Study Population

Predefined nonresponse, not-applicable, and invalid-response codes specified in the survey codebook were treated as missing before analysis. For gaming-time variables, the code indicating no gaming (88) was recoded as 0, whereas predefined missing codes were converted to missing values (NA). Participants were not required to have complete observations across all three assessment waves. The linear mixed-effects models were estimated using maximum likelihood and included available observations with observed CDI scores and complete data on the predictors required for each model. Thus, participants with incomplete follow-up could contribute their available observations, resulting in slight variations in analytic sample sizes across analyses. The likelihood-based handling of missing follow-up outcomes relied on the missing-at-random assumption; missing CDI outcomes were not imputed. For the machine learning analyses, available observations with valid CDI outcomes were included, while missing values in continuous and categorical predictors were imputed using the median and the most frequent category, respectively, within each training fold of the cross-validation pipeline. After excluding observations with missing values in variables required for Model A, the primary analytic sample comprised 2194 participants contributing 6267 observations; Models B and C used the same observations. The machine learning analyses used all 6583 observations with valid CDI scores from the 2294 participants, with missing predictor values imputed within the cross-validation pipeline. Sample sizes for the additional analyses are reported in Section 3.2. Detailed coding rules for invalid and missing responses are provided in Table S1.

#### 2.3.2. Age Computation Across Waves

Age at each assessment wave was calculated from each participant’s date of birth and a wave-specific reference date for the baseline, 12-month, and 24-month assessments (Supplementary Materials S2).

#### 2.3.3. Long-Format Conversion

The original wide-format dataset was converted to a long-format structure (participant × time) for repeated-measures analyses. Time-specific variables were harmonized across assessment waves. Assessment wave was coded as 0, 1, and 2 for the baseline, 12-month, and 24-month assessments, respectively, and was modeled as a continuous linear term. Observations with missing CDI outcome values were excluded.

#### 2.3.4. Variable Types and Centering

Continuous variables were treated as numeric, and categorical variables were converted to factors. To improve model interpretability and facilitate the estimation of interaction effects, time and continuous predictor variables were grand-mean centered before analysis. For the fitted models, the wave variable was centered as time_c = time–mean(time), where mean(time) was calculated from the observations included in the corresponding analytic dataset. Unless otherwise specified for a model-specific parameterization, continuous predictors were centered by subtracting their respective means within the corresponding analytic dataset.

### 2.4. Statistical Analysis: Linear Mixed-Effects Models (LMM)

#### Random Effects Structure and Estimation

To account for repeated measurements within participants, linear mixed-effects models with random intercepts and random slopes for time were fitted using maximum likelihood estimation. When convergence problems occurred, the models were simplified to random-intercept-only specifications (Supplementary Materials S3).

### 2.5. Model Specifications

IGUESS, gaming time, and CDI were measured concurrently at each assessment wave. Accordingly, the mixed-effects models estimated within-wave associations across repeated observations while accounting for within-participant correlation; they were not designed to estimate prospective or directional associations between earlier gaming-related measures and subsequent depressive symptoms. All models used the same covariates and subject-specific random-effects structure, including a random intercept and random slope for time.

#### 2.5.1. Model A: Basic Repeated-Measures LMM

The model simultaneously included the main effects of time, IGUESS, and log-transformed gaming time, together with their interactions with time to examine whether their associations with depressive symptoms varied across assessment waves. The model was adjusted for age, SCHOST, SES, SSAS, PHQ-9, sex, ELPR, JOBPR, MAST, and LIFELV. A subject-specific random intercept and random slope for time were included to account for repeated observations within participants and individual differences in trajectories over time. Detailed variable construction and model specifications are provided in Supplementary Materials S3.

#### 2.5.2. Model B: Two-Component Parameterization of Gaming-Time Exposure LMM

To represent the zero-heavy distribution of gaming-time exposure, gaming behavior was decomposed into gaming participation (any_game) and gaming intensity among participants who engaged in gaming (weekly_pos). Gaming participation was represented by a binary indicator, with any_game = 0 indicating no gaming during the week and any_game = 1 indicating any gaming during the week. The weekly_pos variable was log-transformed as log(1 + weekly_pos) to reduce right skewness before being included in the model. Among observations with positive gaming time, the log-transformed values were centered around the mean calculated from gaming observations only, while observations with no gaming were excluded from the centering procedure and retained a value of 0. Thus, the participation component distinguished observations with and without gaming, whereas the intensity component represented variation in gaming time among observations with positive gaming time.

#### 2.5.3. Model C: Within–Between Decomposition (Person-Mean Centering)

For IGUESS and log-transformed gaming time, person-level means and within-person deviations were derived to distinguish between-person differences from within-person changes over time. The between-person component was defined as each participant’s mean value across available assessment waves, whereas the within-person component was defined as the deviation of each observation from that participant’s mean. Both components were simultaneously included in the model to estimate between-person and within-person associations with depressive symptoms.

### 2.6. Moderation and Stratified Analyses

Moderation analyses evaluated interactions between IGUESS and sex, age at first gaming use, and nighttime gaming use. Additional extension models examined content diversity (GMEP) and parental intervention (ONGR) as complementary baseline factors. Stratified analyses were also conducted by refitting the primary model within each level of categorical moderators when subgroup sample sizes were sufficiently large (e.g., at least 50 participants).

### 2.7. Sensitivity Analyses

Two sensitivity analyses were conducted to assess the robustness of the findings. First, a gaming-time-only exposure model was fitted in which all IGUESS-related terms were removed, to examine the association of gaming time with CDI scores without adjustment for problematic gaming symptoms. Second, to assess whether the association with gaming intensity was sensitive to the treatment of zero gaming time, Model A was refitted after restricting the sample to observations with positive gaming time (>0 min).

### 2.8. Additional Analyses: Change-Score (Δ) Mixed-Effects Model

An additional change-score (Δ) mixed-effects model examined whether within-interval changes in IGUESS and log-transformed gaming time were associated with changes in CDI across the baseline–12-month, 12–24-month, and baseline–24-month intervals. Models adjusted for CDI at the beginning of each interval, interval length and type, and the baseline covariates included in the primary analyses and incorporated participant-specific random intercepts. Detailed variable construction and model specifications are provided in Supplementary Materials S3.

### 2.9. Machine Learning Analyses

To complement the linear mixed-effects models, machine learning–based binary classification analyses were conducted to classify concurrent elevated depressive symptoms (Supplementary Materials S4). Elevated depressive symptoms were defined as a CDI score ≥ 22 [22], and the predictors included in Model A (gaming-related variables together with the demographic, socioeconomic, and psychosocial covariates) were used for classification.

Three classifiers were selected to represent complementary modeling approaches: Logistic Regression as an interpretable linear benchmark, Random Forest as a bagging-based ensemble capable of capturing nonlinear relationships and interactions, and XGBoost as a gradient-boosting method for modeling complex patterns in structured tabular data. The analysis was intended to compare classification performance across models of differing complexity rather than to develop a deployment-ready clinical screening tool. Continuous variables were imputed and standardized, whereas categorical variables were imputed and one-hot encoded. Class imbalance was addressed within hyperparameter tuning by including class-weighting parameters (class_weight for Logistic Regression and Random Forest; scale_pos_weight for XGBoost) in the search space.

Model performance was evaluated using accuracy, ROC-AUC, average precision (AP), precision, recall, F1-score, specificity, negative predictive value (NPV), and the numbers of true-positive (TP) and false-positive (FP) classifications per 1000 observations. Hyperparameter tuning and model evaluation were performed using a nested cross-validation framework while maintaining participant-level separation to prevent information leakage across repeated observations. The outer cross-validation consisted of 5-fold stratified group cross-validation repeated 10 times and was used to estimate model performance. Within each outer training fold, hyperparameters were optimized using an inner 5-fold stratified group cross-validation procedure, with participant identifiers (SUBJNO) used as the grouping variable throughout both the inner and outer cross-validation loops. Hyperparameter combinations were evaluated using average precision (AP) as the optimization metric. For logistic regression, all prespecified hyperparameter combinations were evaluated, whereas randomized search was used for Random Forest and XGBoost, with 30 candidate combinations evaluated for each model. After selecting the optimal hyperparameters within each outer training fold, the classification threshold was determined using out-of-fold predictions generated from the inner cross-validation. Consistent with the sensitivity-prioritized objective of the classification analysis, the threshold that maximized precision among thresholds achieving a recall of at least 0.75 was selected. The model was then refitted on the entire outer training fold using the selected hyperparameters and evaluated on the corresponding outer test fold using the threshold determined exclusively from the training data. This procedure ensured that both hyperparameter optimization and threshold selection were conducted independently of the outer test data. Model interpretation was based on regression coefficients for Logistic Regression and feature importance for the tree-based models. Analyses were conducted in the overall sample and in sex-stratified subgroups.

### 2.10. Ethics Approval

The present study was a secondary analysis of de-identified cohort data from the Cohort Study of Internet/Game/Smartphone Users, established and managed by the National Center for Mental Health (NCMH), Republic of Korea. The authors did not directly recruit, contact, or collect data from the study participants.

Access to the dataset was granted by the NCMH following its formal data-use review and approval process (Data Use Approval No. CSIG-12). In addition, the present study was determined to be exempt from IRB review by the Institutional Review Board of The Catholic University of Korea (Protocol No. 1040395-202508-909) because it involved secondary analysis of de-identified data.

### 2.11. Software

Data preprocessing and statistical analyses were conducted in a Python 3.11.9 environment. Data handling and variable generation were performed using pandas (version 2.3.1) and NumPy (version 2.2.4). Linear mixed-effects models were fitted using statsmodels (version 0.14.6; MixedLM). Logistic Regression and Random Forest models were implemented using scikit-learn (version 1.8.0), and XGBoost classification was implemented using XGBClassifier from the xgboost package (version 3.1.2). Machine learning preprocessing, including missing-value imputation, standardization, and one-hot encoding, was implemented using scikit-learn’s Pipeline and ColumnTransformer frameworks. Nested cross-validation and hyperparameter tuning were implemented using StratifiedGroupKFold and RandomizedSearchCV, respectively. Model performance, including ROC-AUC, average precision (AP), precision, recall, and F1-score, as well as threshold selection based on precision–recall curves, was evaluated using scikit-learn.metrics. Figures and visualizations were generated using matplotlib (version 3.10.1).

## 3. Results

### 3.1. Baseline Characteristics of Participants

A total of 2294 participants were included in the analysis, comprising 1307 males (57.0%) and 987 females (43.0%). At baseline, most participants, regardless of sex, had parents with at least a college-level education (66.2%), had employed parents (66.0%), reported a moderate living standard (50.7%), and lived in two-parent households (92.5%). The mean School Bullying Scale (SCHOST), SES, and SSAS scores were 13.34 ± 2.67, 31.09 ± 5.52, and 96.85 ± 16.25, respectively. Only a small proportion of participants had major depressive disorder (MDD) at baseline (0.1%), and parental depressive symptom scores measured using the PHQ-9 were generally low (mean, 2.41 ± 3.16).

Sex-stratified comparisons showed that females had significantly higher mean CDI scores (9.26 ± 7.25) and GMEP total scores (5.13 ± 1.69) than males (both *p* < 0.001). In contrast, males had significantly higher mean IGUESS scores (4.12 ± 3.99) and weekly gaming time (783.48 ± 690.01 min) than females (both *p* < 0.001). In addition, the proportion classified as nighttime gamers was higher among males (49.5%; *n* = 547) than among females (35.4%; *n* = 291) (Table 1).

### 3.2. Study Population

The processed dataset included 2294 participants contributing 6583 observations across three assessment waves. Of the 2294 participants included at baseline, 2184 (95.2%) and 2105 (91.8%) were retained at the 12- and 24-month follow-ups, respectively, and 2093 (91.2%) completed all three waves. Participants who did not complete all three assessment waves (*n* = 201) were classified as having incomplete follow-up. Among these 201 participants, 12 were missing the 12-month assessment only, 91 were missing the 24-month assessment only, and 98 were missing both follow-up assessments. Compared with participants who completed all three waves, those with incomplete follow-up had higher baseline CDI scores (9.49 ± 8.32 vs. 8.12 ± 6.95, SMD = 0.194), school bullying scores (13.86 ± 3.57 vs. 13.29 ± 2.56, SMD = 0.215), and IGUESS scores (4.15 ± 4.39 vs. 3.39 ± 3.80, SMD = 0.197), whereas the remaining continuous baseline characteristics showed smaller standardized differences and categorical characteristics did not differ significantly between groups. Further details of the attrition analysis are provided in Supplementary Materials S5, including the comparison of baseline characteristics presented in Table S2. Model A included 2194 participants contributing 6267 observations after excluding observations with missing values in the required model variables for required variables. Models B and C used the same analytic sample because both were derived from the complete-case dataset used for Model A.

Additional analyses requiring model-specific variables resulted in further reductions in sample size. Analyses including age at first use, nighttime gaming group, GMEP, and ONGR included 2169, 1849, 2188, and 2180 participants, respectively. The change-score (Δ) analysis required complete repeated observations for calculating change scores and therefore included 2061 participants.

### 3.3. Linear Mixed-Effects Models

#### 3.3.1. Model A: Basic Repeated-Measures LMM

Problematic gaming behavior, as measured by IGUESS, was significantly associated with higher CDI scores, whereas gaming time and all interactions with time were not statistically significant (Table 2).

#### 3.3.2. Model B: Two-Component Parameterization of Gaming-Time Exposure LMM

After separating gaming participation from gaming intensity, IGUESS remained significantly associated with CDI scores. Neither gaming participation nor gaming intensity was significantly associated with CDI scores. No interactions with time were statistically significant (Table 2).

#### 3.3.3. Model C: Within–Between Decomposition

Higher IGUESS scores were concurrently associated with higher CDI scores at both the between-person and within-person levels. In contrast, only the between-person association of gaming time was significant, whereas within-person changes in gaming time and interactions with time were not (Table 2).

### 3.4. Moderation and Stratified Analyses

No significant interaction was observed. IGUESS remained significant across all subgroup analyses (Table 3).

### 3.5. Sensitivity Analyses

In the gaming-time-only sensitivity analysis, log-transformed gaming time was positively and significantly associated with CDI (β = 0.113, *p* < 0.001), in contrast to the null or negative estimates observed in the primary models that included IGUESS. In the positive-gametime-only sensitivity analysis, IGUESS remained positively and significantly associated with CDI (β = 0.427, *p* < 0.001), whereas log-transformed gaming time was not significantly associated with CDI (β = −0.017, *p* = 0.843) (Table 4).

### 3.6. Additional Analyses: Change-Score (Δ) Mixed-Effects Model

Changes in IGUESS, but not gaming time, were significantly associated with changes in CDI. Interval length, CDI at the start of the interval, and age at the start of the interval were also significant predictors of CDI change (Table 4).

### 3.7. Sex-Specific Trajectories Across Assessment Waves

**Figure 1:**
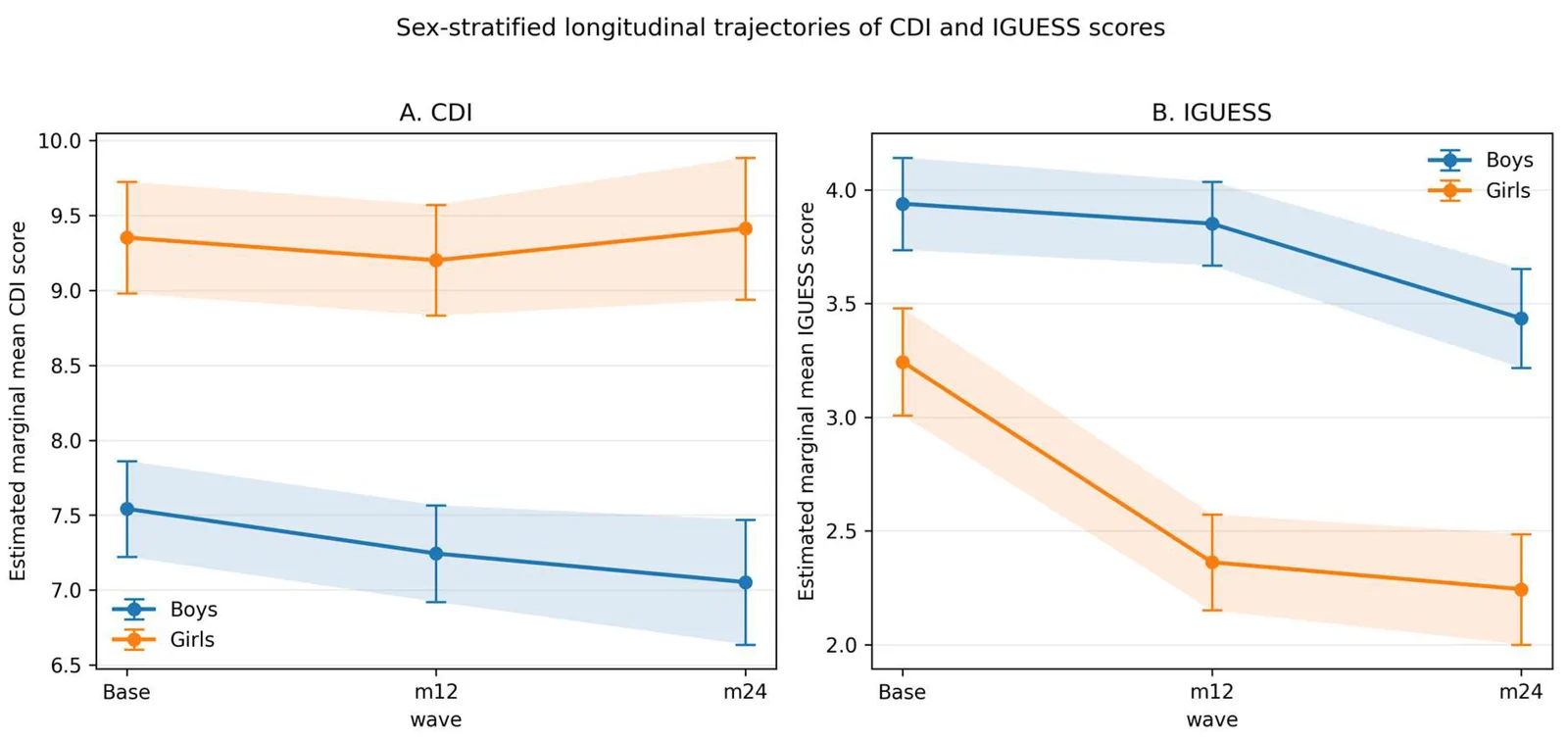
Mean CDI and IGUESS scores by sex across the three assessment waves. Error bars indicate 95% confidence intervals. Trajectories are presented for descriptive purposes; formal tests of change over time are reported in Table 2.

Because CDI and IGUESS scores differed by sex (*p* < 0.001), mean trajectories across the three waves were plotted separately for boys and girls (Figure 1). Both groups showed only minor wave-to-wave fluctuation, with no consistent directional trend over 24 months. These trajectories are descriptive and were not formally tested. In the mixed-effects models, the time × IGUESS interactions were not statistically significant across Models A–C (Table 2). Furthermore, the IGUESS × sex interaction was not significant (β = 0.047, *p* = 0.279), with similar IGUESS–CDI associations observed in boys (β = 0.426) and girls (β = 0.412) (Table 3).

### 3.8. Machine Learning Analyses

Overall, the three models showed similar ROC-AUC (0.870–0.872) and AP (0.410–0.425) values. At the sensitivity-prioritized thresholds, recall ranged from 0.741 to 0.752, whereas precision (0.198–0.220) and F1-scores (0.312–0.339) remained relatively low. Specificity ranged from 0.821 to 0.844, while NPV was consistently high (0.982–0.983). At these thresholds, the models yielded approximately 41–42 true-positive and 147–169 false-positive classifications per 1000 observations (Table S3, Supplementary Materials S6).

#### Sex-Stratified Analyses

The proportion of observations with elevated depressive symptoms (CDI ≥ 22) was 5.6% (368/6583). In sex-stratified analyses, this proportion was higher among females (7.3%; 208/2843) than among males (4.3%; 160/3740). In the male subgroup, ROC-AUC ranged from 0.867 to 0.881 and AP from 0.369 to 0.423, whereas precision (0.178–0.197) and F1-scores (0.285–0.309) remained relatively low. Recall ranged from 0.732 to 0.739, with specificity ranging from 0.846 to 0.864 and NPV from 0.986 to 0.987. In the female subgroup, ROC-AUC ranged from 0.849 to 0.857 and AP from 0.416 to 0.447, whereas precision (0.202–0.230) and F1-scores (0.316–0.349) remained relatively low. Recall ranged from 0.737 to 0.751, with specificity ranging from 0.765 to 0.802 and NPV from 0.974 to 0.975 (Table 5).

## 4. Discussion

### 4.1. Main Findings

This study examined associations among gaming use patterns, problematic gaming behavior (IGUESS), and depressive symptoms (CDI) in children and adolescents using three-wave cohort data and multiple analytical approaches. The principal findings were as follows. First, across the primary linear mixed-effects models, higher IGUESS scores were consistently associated with higher CDI scores (Models A–C, all *p* < 0.001). In contrast, associations with gaming time were model-dependent: gaming time was positively associated with CDI when IGUESS was excluded but was attenuated to null or negative estimates when IGUESS was included. Second, within–between decomposition showed that both higher between-person mean IGUESS levels and within-person deviations in IGUESS were significantly associated with higher CDI scores (both associations, *p* < 0.001). Third, moderation analyses by sex, age at first gaming use, and nighttime gaming status showed that the association between IGUESS and CDI was generally similar across subgroups, with no statistically significant interaction effects. These findings suggest that the association between problematic gaming behavior and depressive symptoms may be relatively consistent across these characteristics rather than confined to specific subgroups. Finally, the complementary machine learning–based concurrent classification analyses yielded ROC-AUC values of 0.870–0.872 and AP values of 0.410–0.425 in the overall sample. At the sensitivity-prioritized thresholds, recall ranged from 0.741 to 0.752 and specificity from 0.821 to 0.844, whereas precision and F1-scores ranged from 0.198 to 0.220 and from 0.312 to 0.339, respectively. NPV was consistently high, ranging from 0.982 to 0.983. Precision and F1-scores were also modest in the sex-stratified analyses, indicating a substantial false-positive burden and supporting cautious interpretation of the classification results with respect to potential real-world application.

### 4.2. Interpretation and Discussion

The findings showed consistent associations between problematic gaming symptoms and depressive symptoms across models, whereas associations with gaming time were more dependent on model specification. Whereas associations with gaming time may vary depending on contextual factors [23], problematic gaming indicators may more directly reflect functional impairment and behavioral dependence related to gaming [24]. This interpretation is consistent with previous reports linking problematic gaming with mental health problems [25,26], including depression, anxiety, and impaired social functioning. The present study extends this literature by jointly modeling gaming time and problematic gaming symptoms across repeated assessment waves and by distinguishing within-person associations from stable between-person differences. The within-person findings are particularly relevant because they indicate that the association was not explained solely by stable differences between individuals. Higher IGUESS scores relative to an individual’s own average were also associated with higher depressive symptom scores at the same assessment periods. This suggests that fluctuations in problematic gaming symptoms may track fluctuations in depressive symptoms within the same individual over time. However, because both measures were assessed concurrently at each wave, the direction of this relationship cannot be determined.

Notably, the association between gaming time and depressive symptoms depended on whether problematic gaming symptoms were included in the model. Gaming time was positively associated with CDI scores when modeled without IGUESS (β = 0.113, *p* < 0.001), whereas the corresponding estimates were attenuated to null or negative values when IGUESS was simultaneously included. Given the conceptual and empirical overlap between gaming time and problematic gaming symptoms, this pattern may reflect shared variance between the two exposures and suggests that estimates for gaming time were sensitive to model specification. The negative adjusted coefficients should not be interpreted as indicating a protective effect of gaming time; rather, conditional on IGUESS and the other model covariates, greater gaming time did not show a consistent positive association with depressive symptoms. Because gaming behavior, IGUESS, and CDI were assessed concurrently at each wave, this pattern should not be interpreted as evidence of mediation or a causal pathway.

The machine learning analyses were conducted as complementary concurrent classification analyses rather than as models for prospective prediction or standalone screening. Although ROC-AUC values were relatively high, AP, precision, and F1-scores showed more modest performance, particularly at the sensitivity-prioritized thresholds. Recall was approximately 0.74–0.75, while specificity ranged from 0.821 to 0.844 and NPV remained high at 0.982–0.983 in the overall sample. Despite the high NPV, the modest precision was accompanied by a substantial number of false-positive classifications relative to true-positive classifications, as reflected in the numbers of true-positive and false-positive classifications per 1000 observations. Such a false-positive burden could limit the practical utility of these models and may contribute to unnecessary follow-up assessments or alarm fatigue if applied as standalone screening tools. Accordingly, these findings should be interpreted as complementary concurrent classification results rather than as evidence of suitability for standalone screening.

### 4.3. Limitations and Future Directions

This study has several limitations. First, gaming behavior and depressive symptoms were assessed using self-reported questionnaires and may therefore be subject to response bias and measurement error. Second, although the data were collected longitudinally, the primary exposures and outcome were measured concurrently at each wave. Therefore, the estimated associations represent co-occurring differences and changes across repeated assessments and should not be interpreted as prospective prediction or evidence of temporal directionality. Future studies should consider approaches such as random-intercept cross-lagged panel models or causal inference methods to clarify directionality. In addition, although 91.2% of participants completed all three waves, those with incomplete follow-up had modestly higher baseline depressive symptoms, problematic gaming symptoms, and school bullying scores (standardized mean differences approximately 0.2). The mixed-effects analyses included available observations with complete data on the required model variables and relied on a missing-at-random assumption for missing follow-up outcomes. Nevertheless, bias related to missing data and selective attrition cannot be fully excluded. Third, because the study used a school-based South Korean cohort, generalizability to other populations and cultural contexts may be limited. Korea-specific cultural and parenting characteristics may have influenced both gaming behavior and depressive symptoms; replication in cohorts from other countries and cultural settings is therefore warranted. Fourth, the machine learning analyses evaluated concurrent classification rather than temporal prediction because predictors and depression outcomes were measured at the same assessment waves. Accordingly, these findings should not be interpreted as forecasting future depression onset. Fifth, although the classification models were evaluated using repeated nested cross-validation with systematic hyperparameter tuning, they were not externally validated in an independent cohort. Given the low prevalence of elevated depressive symptoms, precision remained limited despite high ROC-AUC values, and performance may therefore be optimistic relative to real-world settings. Future studies should develop temporally predictive models using baseline predictors and subsequent outcomes, incorporating external validation in independent cohorts. Further longitudinal research should also incorporate a broader range of psychosocial and environmental factors and examine long-term changes in gaming behavior and their relationships with mental health outcomes.

## 5. Conclusions

This study examined the concurrent associations of problematic gaming behavior and gaming time with depressive symptoms in children and adolescents. Problematic gaming behavior was consistently associated with concurrent depressive symptoms across multiple mixed-effects models, at both the between-person and within-person levels. In contrast, associations with gaming time were less consistent and varied according to whether problematic gaming symptoms were considered in the model.

These findings suggest that problematic gaming symptoms may provide a more consistent indicator of depressive symptoms than gaming time alone in children and adolescents. Because the primary analyses examined concurrent associations, further research is needed to clarify the temporal and directional relationships among gaming time, problematic gaming symptoms, and depressive symptoms and to determine their implications for mental health assessment in children and adolescents.

## Figures and Tables

**Table 1:** Baseline characteristics of the study participants.

Variable	Total	Boys	Girls	*p* Value
Demographic characteristics				
Sex, *n* (%)	2294	1307 (57.0)	987 (43.0)	
Age (years), mean ± SD	12.86 ± 1.21	12.90 ± 1.18	12.81 ± 1.24	0.0973
Socioeconomic characteristics				
Parental education level (ELPR) *, *n* (%)				0.4338
Low	773 (33.8)	431 (33.1)	342 (34.8)	
High	1513 (66.2)	871 (66.9)	642 (65.2)	
Missing, *n*	8	5	3	
Parental occupation status (JOBPR), *n* (%)				0.4952
Employed	1504 (66.0)	867 (66.6)	637 (65.1)	
Unemployed/Other	776 (34.0)	435 (33.4)	341 (34.9)	
Missing, *n*	14	5	9	
Parental marital status (MAST), *n* (%)				0.7575
Two-parent family	2121 (92.5)	1206 (92.3)	915 (92.7)	
Other (single/divorced/widowed)	173 (7.5)	101 (7.7)	72 (7.3)	
Parental living standard (LIFELV) †, *n* (%)				0.9956
Low	411 (18.6)	235 (18.7)	176 (18.6)	
Middle	1117 (50.7)	636 (50.6)	481 (50.8)	
High	676 (30.7)	386 (30.7)	290 (30.6)	
Missing, *n*	90	50	40	
Baseline mental health				
Baseline major depressive disorder (MDD), *n* (%)				0.8076
Yes	3 (0.1)	1 (0.1)	2 (0.2)	
No	2290 (99.9)	1305 (99.9)	985 (99.8)	
Missing, *n*	1	1	0	
PHQ-9 score, mean ± SD	2.41 ± 3.16	2.41 ± 3.27	2.41 ± 3.01	0.9892
Missing, *n*	6	2	4	
CDI score, mean ± SD	8.24 ± 7.09	7.47 ± 6.86	9.26 ± 7.25	<0.001
Psychosocial factors				
School Bullying score (SCHOST), mean ± SD	13.34 ± 2.67	13.25 ± 2.79	13.45 ± 2.49	0.0723
Self-Esteem Scale score (SES), mean ± SD	31.09 ± 5.52	31.41 ± 5.32	30.65 ± 5.74	0.0013
Social Support Appraisals Scale score (SSAS), mean ± SD	96.85 ± 16.25	96.05 ± 16.76	97.90 ± 15.49	0.0063
GMEP total score, mean ± SD	4.52 ± 1.89	4.06 ± 1.90	5.13 ± 1.69	<0.001
Missing, *n*	9	5	4	
Gaming and media use				
Night-time gaming use, *n* (%)				<0.001
No	1091 (56.6)	559 (50.5)	532 (64.6)	
Yes	838 (43.4)	547 (49.5)	291 (35.4)	
Missing, *n*	365	201	164	
Weekly game time (minutes), mean ± SD	615.82 ± 693.09	783.48 ± 690.01	393.14 ± 632.08	<0.001
Missing, *n*	31	16	15	
Log-transformed game time, mean ± SD	5.06 ± 2.59	6.01 ± 1.76	3.79 ± 2.95	<0.001
Missing, *n*	31	16	15	
IGUESS score, mean ± SD	3.46 ± 3.86	4.12 ± 3.99	2.58 ± 3.49	<0.001
Age at first use, mean ± SD	3.53 ± 2.02	3.37 ± 1.72	3.74 ± 2.34	<0.001
Missing, *n*	28	2	26	

Data are presented as *n* (%) for categorical variables and mean ± SD for continuous variables. Percentages are based on participants with valid responses. * Parental education level (ELPR): High indicates college entrance or higher, whereas Low indicates high school graduation or lower. † Parental living standard (LIFELV): Parents rated their household living standard as low, middle, or high without predefined objective criteria. Abbreviations: CDI, Children’s Depression Inventory; ELPR, parental education level; GMEP, Game Motivation and Engagement Problems; IGUESS, Internet Game Use-Elicited Symptom Screen; JOBPR, parental occupation status; LIFELV, parental living standard; MAST, parental marital status; MDD, major depressive disorder; PHQ-9, Patient Health Questionnaire-9; SD, standard deviation; SSAS, Social Support Appraisals Scale.

**Table 2:** Associations of gaming-related variables with CDI scores in the primary repeated-measures mixed-effects models.

Variable	Model A β (SE)	95% CI	*p* Value	Model B β (SE)	95% CI	*p* Value	Model C β (SE)	95% CI	*p* Value
IGUESS	0.411 (0.021)	[0.369, 0.453]	<0.001	0.410 (0.022)	[0.367, 0.453]	<0.001			
IGUESS between-person							0.449 (0.036)	[0.379, 0.519]	<0.001
IGUESS within-person							0.393 (0.026)	[0.341, 0.444]	<0.001
Game time (log)	−0.049 (0.032)	[−0.112, 0.013]	0.119						
Any game use				−0.318 (0.201)	[−0.712, 0.075]	0.113			
Weekly positive game time (log)				−0.038 (0.083)	[−0.201, 0.125]	0.650			
Game time between-person (log)							−0.128 (0.057)	[−0.239, −0.017]	0.024
Game time within-person (log)							−0.022 (0.038)	[−0.096, 0.053]	0.568
Time	−0.125 (0.106)	[−0.334, 0.083]	0.239	−0.058 (0.198)	[−0.446, 0.330]	0.770	−0.124 (0.106)	[−0.332, 0.085]	0.245
Time × IGUESS	0.013 (0.022)	[−0.030, 0.056]	0.560	0.007 (0.023)	[−0.037, 0.051]	0.752			
Time × IGUESS (within)							0.026 (0.038)	[−0.049, 0.101]	0.501
Time × Game time (log)	−0.005 (0.031)	[−0.067, 0.056]	0.862						
Time × Any game use				−0.092 (0.204)	[−0.492, 0.308]	0.652			
Time × Weekly positive game time (log)				0.088 (0.091)	[−0.090, 0.266]	0.334			
Time × Game time (log, within)							−0.071 (0.056)	[−0.181, 0.040]	0.209

Model A: Basic repeated-measures linear mixed-effects model. Model B: Two-component parameterization of gaming-time exposure linear mixed-effects model including gaming participation and weekly gaming intensity among participants who engaged in gaming. Model C: Within–between decomposition model separating between-person and within-person effects. All models were adjusted for age, sex, parental education, parental occupation, socioeconomic status, bullying (SCHOST), self-esteem (SES), social support (SSAS), and parental depressive symptoms (PHQ-9).

**Table 3:** Moderation and stratified analyses of the association between IGUESS and CDI scores.

Model/Variable	β	SE	95% CI	*p* Value
IGUESS × SEX moderation model				
IGUESS × SEX	0.047	0.043	[−0.038, 0.132]	0.279
Sex-stratified models				
IGUESS (Male)	0.426	0.025	[0.377, 0.476]	<0.001
IGUESS (Female)	0.412	0.040	[0.334, 0.489]	<0.001
IGUESS × age at first use moderation model				
IGUESS × age at first use	−0.011	0.011	[−0.031, 0.010]	0.319
Age at first use stratified models				
IGUESS (Early age at first use)	0.435	0.028	[0.380, 0.490]	<0.001
IGUESS (Late age at first use)	0.375	0.033	[0.310, 0.440]	<0.001
IGUESS × nighttime group moderation model				
IGUESS × nighttime group	0.016	0.043	[−0.069, 0.100]	0.717
Nighttime group stratified models				
IGUESS (Nighttime group = 0)	0.392	0.032	[0.329, 0.454]	<0.001
IGUESS (Nighttime group = 1)	0.417	0.035	[0.350, 0.485]	<0.001
GMEP exposure model				
GMEP	0.132	0.057	[0.021, 0.243]	0.020
GMEP + IGUESS model				
IGUESS	0.411	0.022	[0.369, 0.453]	<0.001
GMEP	0.087	0.055	[−0.020, 0.194]	0.112
ONGR model				
Parental intervention (ONGR)	−0.100	0.051	[−0.200, 0.001]	0.053
IGUESS	0.411	0.022	[0.369, 0.453]	<0.001

Moderation models evaluated whether the association between IGUESS and CDI differed according to subgroup characteristics. Stratified models were fitted separately within each subgroup when the sample size was sufficient. ONGR, Parental intervention level.

**Table 4:** Additional and sensitivity analyses of associations with CDI scores.

Model/Variable	β	SE	95% CI	*p* Value
Δ-change mixed-effects model				
ΔIGUESS	0.216	0.017	[0.183, 0.249]	<0.001
Δlog gametime	−0.024	0.024	[−0.070, 0.023]	0.315
Δt (time interval)	0.319	0.101	[0.121, 0.517]	0.002
CDI at start of interval	−0.882	0.016	[−0.913, −0.850]	<0.001
Age at start of interval	0.364	0.073	[0.222, 0.506]	<0.001
Sensitivity analysis: gametime-only exposure model				
log gametime	0.113	0.031	[0.052, 0.175]	<0.001
Time	−0.275	0.109	[−0.489, −0.060]	0.012
Time × log gametime	0.007	0.030	[−0.051, 0.065]	0.822
Sensitivity analysis: positive-gametime-only model				
IGUESS	0.427	0.023	[0.383, 0.471]	<0.001
log gametime	−0.017	0.086	[−0.186, 0.152]	0.843
Time	−0.199	0.114	[−0.424, 0.025]	0.081
Time × IGUESS	0.010	0.023	[−0.035, 0.056]	0.658
Time × log gametime	0.122	0.091	[−0.057, 0.300]	0.181

The change-score (Δ) mixed-effects model examined whether changes in IGUESS and gaming time were associated with changes in CDI across intervals. gametime-only exposure model evaluated the gaming-time association after removal of IGUESS-related terms. positive-gametime-only model evaluated the association between gaming intensity and CDI using only observations with gaming time > 0 min.

**Table 5:** Performance of machine learning models for concurrent classification of elevated depressive symptoms in the overall sample and sex-stratified subgroups.

Analysis Group	Model	Accuracy	ROC-AUC	AP	Recall	Precision	F1-Score	Specificity	NPV
Overall	Logistic Regression	0.838 ± 0.015 [0.826, 0.851]	0.872 ± 0.025 [0.849, 0.891]	0.425 ± 0.061 [0.368, 0.490]	0.741 ± 0.070 [0.688, 0.783]	0.220 ± 0.019 [0.193, 0.249]	0.339 ± 0.026 [0.301, 0.375]	0.844 ± 0.018 [0.831, 0.857]	0.982 ± 0.005 [0.978, 0.986]
	Random Forest	0.817 ± 0.021 [0.804, 0.830]	0.870 ± 0.023 [0.849, 0.890]	0.415 ± 0.065 [0.361, 0.483]	0.741 ± 0.071 [0.694, 0.786]	0.198 ± 0.018 [0.174, 0.224]	0.312 ± 0.025 [0.278, 0.345]	0.821 ± 0.024 [0.807, 0.834]	0.982 ± 0.005 [0.978, 0.986]
	XGBoost	0.823 ± 0.019 [0.810, 0.836]	0.870 ± 0.022 [0.846, 0.889]	0.410 ± 0.063 [0.352, 0.476]	0.752 ± 0.069 [0.701, 0.799]	0.206 ± 0.018 [0.179, 0.235]	0.322 ± 0.024 [0.286, 0.359]	0.827 ± 0.023 [0.813, 0.841]	0.983 ± 0.004 [0.979, 0.986]
Male	Logistic Regression	0.858 ± 0.023 [0.842, 0.874]	0.881 ± 0.033 [0.845, 0.907]	0.423 ± 0.089 [0.345, 0.507]	0.736 ± 0.098 [0.654, 0.799]	0.197 ± 0.024 [0.155, 0.238]	0.309 ± 0.030 [0.251, 0.359]	0.864 ± 0.026 [0.847, 0.880]	0.987 ± 0.005 [0.982, 0.990]
	Random Forest	0.857 ± 0.022 [0.840, 0.873]	0.877 ± 0.033 [0.843, 0.905]	0.392 ± 0.086 [0.329, 0.479]	0.739 ± 0.103 [0.667, 0.802]	0.195 ± 0.021 [0.159, 0.234]	0.308 ± 0.030 [0.256, 0.354]	0.862 ± 0.026 [0.845, 0.879]	0.987 ± 0.005 [0.983, 0.991]
	XGBoost	0.841 ± 0.029 [0.826, 0.857]	0.867 ± 0.032 [0.831, 0.898]	0.369 ± 0.085 [0.302, 0.455]	0.732 ± 0.110 [0.658, 0.796]	0.178 ± 0.027 [0.143, 0.215]	0.285 ± 0.037 [0.233, 0.332]	0.846 ± 0.033 [0.829, 0.862]	0.986 ± 0.005 [0.982, 0.990]
Female	Logistic Regression	0.798 ± 0.025 [0.774, 0.818]	0.857 ± 0.026 [0.824, 0.886]	0.447 ± 0.077 [0.365, 0.535]	0.740 ± 0.095 [0.671, 0.802]	0.230 ± 0.025 [0.189, 0.271]	0.349 ± 0.034 [0.294, 0.397]	0.802 ± 0.031 [0.778, 0.824]	0.975 ± 0.008 [0.969, 0.982]
	Random Forest	0.765 ± 0.033 [0.743, 0.787]	0.852 ± 0.024 [0.822, 0.877]	0.432 ± 0.075 [0.346, 0.520]	0.737 ± 0.090 [0.667, 0.796]	0.202 ± 0.023 [0.165, 0.239]	0.316 ± 0.030 [0.266, 0.361]	0.768 ± 0.040 [0.743, 0.791]	0.974 ± 0.008 [0.967, 0.981]
	XGBoost	0.764 ± 0.037 [0.743, 0.786]	0.849 ± 0.025 [0.818, 0.877]	0.416 ± 0.069 [0.340, 0.504]	0.751 ± 0.097 [0.684, 0.808]	0.204 ± 0.024 [0.168, 0.242]	0.319 ± 0.030 [0.269, 0.364]	0.765 ± 0.045 [0.741, 0.789]	0.975 ± 0.008 [0.968, 0.982]

Performance metrics are presented as mean ± standard deviation [95% CI]. Elevated depressive symptoms were defined as CDI ≥ 22. Model performance was evaluated using accuracy, ROC-AUC, AP, recall, precision, F1-score, specificity, and NPV. TP and FP classifications per 1000 observations are presented in Table S3 of Supplementary Materials S6.

## Data Availability

The data supporting the findings of this study were obtained from the school-based Internet, Game, and Smartphone User Cohort established and managed by the National Center for Mental Health (NCMH), Republic of Korea. The data are not publicly available because they contain de-identified participant information and are subject to institutional and ethical restrictions. Access may be granted by the National Center for Mental Health upon reasonable request and approval, in accordance with applicable data-sharing policies and regulations.

## Supplementary Materials

The following supporting information can be downloaded at: https://www.mdpi.com/article/10.3390/healthcare14193315/s1. Supplementary Materials S1, detailed variable definitions and derivations; Table S1, coding and handling of invalid and missing responses; Supplementary Materials S2, detailed data preprocessing; Supplementary Materials S3, detailed mixed-effects model implementation and change-score model specification; Supplementary Materials S4, detailed machine learning procedures; Supplementary Materials S5, attrition analysis, and Table S2, comparison of baseline characteristics between participants with complete and incomplete follow-up; and Supplementary Materials S6, false-positive burden, and Table S3, true-positive and false-positive classifications per 1000 observations.
