# Supplementary material for: Concurrent Associations of Gaming Time and Problematic Gaming with Depressive Symptoms in Children and Adolescents: A Three-Wave Cohort Study with Complementary Machine Learning Classification
Source: Healthcare (Basel). 2026 Oct 5;14(19):3315. doi: 10.3390/healthcare14193315 (PMC13664882; doi:10.3390/healthcare14193315)
Supplement: Supplementary file 1 [file healthcare-14-03315-s001.zip › healthcare-4563076-supplementary.pdf]

Supplementary Materials

Concurrent Associations of Gaming Time and Problematic Gaming with Depressive Symptoms in Children and Adolescents: A Three-Wave Cohort Study with Complementary Machine-Learning Classification

Yeseo Lee, Jun Seok Her and Hyo Jung Kim

Supplementary Materials S1. Detailed Variable Definitions and Derivations

S1.1. Weekly Gaming Time

At each assessment wave, total weekly gaming time was calculated using self-reported weekday and weekend or public-holiday gaming duration. Gaming duration was originally recorded separately in hours and minutes.

Weekday daily gaming time was calculated as:

$$\text{Weekday gaming time (minutes)} = 60 \times \text{GMTWKSH1} + \text{GMTWKSM1}$$

where GMTWKSH1 represents the average number of hours spent gaming per weekday and GMTWKSM1 represents the additional number of minutes spent gaming per weekday.

Weekend or public-holiday daily gaming time was calculated as:

$$\text{Weekend gaming time (minutes)} = 60 \times \text{GMTWKDH1} + \text{GMTWKDM1}$$

where GMTWKDH1 represents the average number of hours spent gaming per weekend day or public holiday and GMTWKDM1 represents the additional number of minutes spent gaming per weekend day or public holiday.

Total weekly gaming time was then calculated as:

$$\text{Weekly gaming time (minutes)} = 5 \times \text{weekday gaming time} + 2 \times \text{weekend gaming time}$$

Total weekly gaming time was restricted to the theoretically possible range of 0–10,080 minutes per week. To reduce right skewness, weekly gaming time was log-transformed as:

$$\text{log\_gametime} = \log(1 + \text{weekly gaming time})$$

A response code of 88, indicating no gaming, was recoded as 0 minutes before weekly gaming time was calculated. Predefined missing or invalid response codes were treated as missing values.

S1.2. Content Diversity (GMEP)

Content diversity was represented by the baseline GMEP total score (base_GMEP_tot). The variable was derived by summing the number of baseline Game Motivation and Engagement Problems (GMEP) items coded as use = 1.

If all corresponding GMEP items were missing for a participant, the derived total score was treated as missing.

This variable was used as an additional baseline factor in extension and moderation analyses.

S1.3. Parental Intervention Level (ONGR)

Parental intervention in children’s gaming behavior was represented by the baseline parental intervention score (base_ongr_tot).

The score was calculated using baseline ONGR1, ONGR2, and ONGR3 items. Responses were recoded as follows:

- Strongly disagree (1) = 0
- Disagree (2) = 1
- Agree (3) = 2
- Strongly agree (4) = 3

The recoded values were summed to generate the parental intervention score.

If all corresponding ONGR items were missing, the derived score was treated as missing.

S1.4. Age at First Gaming Use

Age at first gaming use (base_age_first_use) was derived from four baseline variables:

- base_INTGS
- base_MOBILES
- base_INTGSP
- base_MOBILEGP

Age at first use was defined as the minimum valid value among these four variables.

Because the response categories differed between variables, values 1–3 in base_INTGSP and base_MOBILEGP were recoded to 1, whereas values ≥4 were transformed by subtracting 2 before the minimum value was calculated.

If no valid value was available among the corresponding variables, age at first use was treated as missing.

S1.5. Nighttime Gaming Use

Nighttime gaming use was represented by the baseline nighttime-use group (base_night_group).

Participants were classified as belonging to the nighttime-use group if at least one of the following baseline items was coded as 1:

- IGAT1
- IGAT7
- MGAT1
- MGAT7

Participants meeting this criterion were coded as 1, whereas participants who did not meet the criterion were coded as 0.

If all corresponding items were missing, nighttime-use status was treated as missing.

Table S1. Coding and Handling of Invalid and Missing Responses

Variable or item group	Invalid/nonresponse code(s)	Treatment
CDI total score	99	Missing
IGUESS total score	99	Missing
Parental education level (ELPR)	99	Missing
Parental occupation (JOBPR)	99	Missing
Parental living standard (LIFELV)	99	Missing
Parental PHQ-9	99	Missing
Major depressive disorder (MDD)	9, 99	Missing
INTGS / MOBILES	9, 99	Missing
INTGSP / MOBILEGP	11, 99	Missing
Gaming-time variables	88	Recoded as 0, indicating no gaming
Gaming-time variables	99	Missing
IGAT / MGAT items	8, 9	Missing
ONGR / INTR items	9	Missing

Variable or item group	Invalid/nonresponse code(s)	Treatment
All variables	77777	Missing

All predefined invalid, nonresponse, and not-applicable codes were converted to missing values before descriptive, mixed-effects, and machine-learning analyses. For the mixed-effects models, observations with missing values in variables required for each model were excluded on a model-specific basis; consequently, sample sizes varied slightly across analyses. For the machine-learning analyses, observations with valid CDI outcomes were retained, and missing predictor values were imputed within each training fold (Appendix S4.3).

Supplementary Materials S2. Detailed Data Preprocessing

S2.1. Age Calculation Across Assessment Waves

Participant age at each assessment wave was calculated using the participant's date of birth (BIRTHDT) and a wave-specific reference date.

The reference dates were defined as follows:

- Baseline: January 1, 2016
- 12-month follow-up: January 1, 2017
- 24-month follow-up: January 1, 2018

BIRTHDT was first converted to a date format, after which age was calculated from the difference between the participant's date of birth and the corresponding reference date.

S2.2. Conversion From Wide to Long Format

The original cohort dataset was provided in wide format, with assessment-specific variables stored in separate columns for baseline, 12-month follow-up, and 24-month follow-up.

For repeated-measures analyses, the dataset was converted into a long-format structure in which each row represented a participant at a specific assessment wave.

Wave-specific observations were assigned the following time values:

- Baseline = 0
- 12-month follow-up = 1
- 24-month follow-up = 2

Variables corresponding to each assessment wave were extracted and harmonized to common variable names, including CDI, IGUESS, log_gametime, weekly_minutes, and age.

Observations with missing values for the outcome variable, CDI, were excluded when constructing the repeated-measures analytic dataset.

S2.3. Variable Types and Centering

Continuous variables, including CDI, IGUESS, log-transformed gaming time, weekly gaming time, age, and continuous psychosocial covariates, were treated as numeric variables.

Categorical covariates, including sex, parental education level, parental occupation, parental marital status, and parental living standard, were treated as categorical factors.

To facilitate interpretation of model coefficients and improve numerical stability when estimating interaction terms, time and continuous predictor variables were grand-mean centered before model estimation.

Supplementary Materials S3. Detailed Mixed-Effects Model Implementation

S3.1. Random-Effects Structure and Estimation

Linear mixed-effects models were fitted to account for repeated measurements nested within participants.

The default random-effects structure included both a participant-specific random intercept and a random slope for time.

Models were estimated using maximum likelihood estimation rather than restricted maximum likelihood.

Rank deficiency in the model design matrix was assessed during model construction. When numerical singularity or convergence problems occurred with the random-intercept and random-slope specification, the random slope for time was removed and the model was re-estimated using a random-intercept-only structure.

This fallback procedure was applied to improve model convergence and estimation stability while retaining participant-level clustering.

S3.2. Model A: Basic repeated-measures LMM

The model simultaneously included the main effects of time, IGUESS, and log-transformed gaming time, together with their interactions with time to examine whether their associations with depressive symptoms varied across assessment waves.

$$CDI_{it} = \beta_0 + \beta_1 time_c + \beta_2 IGUESS_c + \beta_3(time_c \times IGUESS_c) + \beta_4 \log\_gametime_c + \beta_5(time_c \times log\_gametime_c) + \beta_6 age_c + \beta_7^{\top} Z_i + u_{0i} + u_{1i} time_c + \epsilon_{it}$$

Here, i denotes the individual participant, and t denotes the assessment wave (baseline = 0, m12 = 1, m24 = 2). CDI_{it} represents the depressive symptom score of individual i at time point t. Z_i represents the vector of baseline covariates, including SCHOST, SES, SSAS, PHQ9, SEX, ELPR, JOBPR, MAST, and LIFELV. u_{0i} denotes the subject-specific random intercept, u_{1i} denotes the subject-specific random slope for time, and ϵ_{it} represents the residual error term.

S3.3. Model B: Two-component parameterization of gaming-time exposure LMM

To examine the association between gaming intensity and depressive symptoms among participants who engaged in gaming, gaming behavior was decomposed into gaming participation (any_game) and gaming intensity among participants who engaged in gaming (weekly_pos).

$$any\_game_{it} = \mathbb{I}(weekly\_minutes_{it} > 0)(0/1)$$

$$weekly\_pos_{it} = weekly\_minutes_{it}$$

$$\text{if } any\_game = 1, \text{else } 0$$

$$\log\_weekly_{pos_{it}} = \log(1 + weekly_{pos_{it}})$$

Gaming participation was represented by a binary indicator of any gaming (any_game), while the gaming intensity variable (weekly_pos) was log-transformed as log(1 + weekly_pos) to reduce right skewness and mean-centered among observations with gaming participation (any_game = 1). Non-gaming observations were excluded from the centering procedure and retained their original value of zero.

$$CDI_{it} = \beta_0 + \beta_1 time_c + \beta_2 IGUESS_c + \beta_3(time_c \times IGUESS_c) + \beta_4 any\_game_{it} + \beta_5(time_c \times any\_game_{it}) + \beta_6 \log\_weekly\_pos_c + \beta_7(time_c \times \log\_weekly\_pos_c) + \beta_8 age_c + \beta_9^{\top} Z_i + u_{0i} + u_{1i} time_c + \epsilon_{it}$$

The definitions of i, t, Z_i, u_{0i}, u_{1i}, and ϵ_{it} are identical to those used in Model A.

S3.4. Model C: Within–between decomposition (person-mean centering)

For IGUESS and log_gametime, person-level means and within-person deviations were derived to distinguish between-person differences from within-person changes over time.

$$IGUESS_{pm,i} = mean_i(IGUESS)$$
$$IGUESS_{wp,it} = IGUESS_{it} - IGUESS_{pm,i}$$
$$logG_{pm,i} = mean_i(logG)$$
$$logG_{wp,it} = logG_{it} - logG_{pm,i}$$
$$CDI_{it} = \beta_0 + \beta_1 time_c + \beta_2 IGUESS_{pm,c} + \beta_3 IGUESS_{wp,c} + \beta_4\left(time_c \times IGUESS_{wp,c}\right) + \beta_5 logG_{pm,c} + \beta_6 logG_{wp,c} + \beta_7(time_c \times logG_{wp,c}) + \beta_8 age_c + \beta_9^{\mathsf{T}} Z_i + u_{0i} + u_{1i} time_c + \epsilon_{it}$$

$IGUESS_{pm,i}$ and $logG_{pm,i}$ represent the person-level means (between-person associations), whereas $IGUESS_{wp,it}$ and $logG_{wp,it}$ represent within-person deviations from each individual's mean (within-person associations).

Accordingly, β_2 and β_5 estimate between-person differences, whereas $\beta_3$and β_6 estimate within-person concurrent associations across repeated assessments within the same individual. The definitions of i, t, Z_i, u_{0i}, u_{1i}, and ϵ_{it} are identical to those used in Model A.

S3.5. Change-Score (Δ) Model: Dataset Construction and Specification

To construct the change-score dataset, the long-format data were sorted chronologically for each participant, and intervals in which two consecutive observations were available for the same individual were extracted. Three interval types were defined: (1) baseline to 12 months (base–m12), (2) 12 months to 24 months (m12–m24), and (3) baseline to 24 months (base–m24).

For each interval, CDI scores at the start and end of the interval were defined as CDI_start and CDI_end, respectively, and the change in depressive symptoms was calculated as ΔCDI = CDI_end − CDI_start. Using the same approach, interval-level changes in IGUESS and gaming-time variables (ΔIGUESS, Δlog_gametime, and additionally Δweekly_minutes) were calculated. Interval length was defined as $\Delta t = t_1 - t_0$ and mean-centered (dt_c) before inclusion in the model.

The dependent variable was ΔCDI, and the primary explanatory variables included dt_c, CDI_start,c, ΔIGUESS_c, Δlog_gametime_c, and age_start,c. Baseline CDI at the start of each interval (CDI_start) was included as a covariate to control for differences in initial symptom severity and potential regression-to-the-mean effects. Interval type (base–m12, m12–m24, and base–m24) was additionally included as a categorical fixed effect to account for systematic differences across interval types. The same baseline covariates used in the primary models were also included, and all continuous explanatory variables were mean-centered to improve interpretability and estimation stability.

Because up to three interval observations could be generated from the same participant, the model was fitted with subject-specific random intercepts to account for within-subject correlations. Observations with missing values in essential model variables (ΔCDI, Δt, CDI_start, ΔIGUESS, Δlog_gametime, age_start, major baseline covariates, and categorical variables) were excluded prior to analysis.

The model was specified as $\Delta CDI\_i,k = \beta_0 + \beta_1 \cdot dt\_c + \beta_2 \cdot CDI\_start,c + \beta_3 \cdot \Delta IGUESS\_c + \beta_4 \cdot \Delta log\_gametime\_c + \beta_5 \cdot age\_start,c + \beta_6^{\mathsf{T}} Z\_i + \gamma^{\mathsf{T}} C(interval\_k) + u\_0i + \varepsilon\_i,k$, where i denotes the individual participant, k denotes the interval type, Z_i represents the vector of baseline covariates, u_0i denotes the participant-specific random intercept, and ε_i,k represents the residual error term.

Supplementary Materials S4. Detailed Machine-Learning Procedures

S4.1. Outcome and Participant-Level Data Splitting

Machine-learning analyses were conducted as concurrent binary classification analyses of elevated depressive symptoms. Elevated depressive symptoms were defined as CDI ≥ 22 (depressed = 1), whereas observations with CDI < 22 were classified as depressed = 0.

Because predictors and CDI outcomes were obtained from the same assessment waves, these analyses were intended to evaluate concurrent discrimination rather than prediction of future depression onset.

To prevent information leakage arising from repeated observations of the same participant appearing in both training and test folds, model development and evaluation were conducted using participant-level nested cross-validation based on the participant identifier (SUBJNO). The outer cross-validation consisted of 5-fold StratifiedGroupKFold repeated 10 times using different random seeds, yielding 50 outer test-fold evaluations for each classifier. All observations from the same participant were assigned exclusively to either the training or test fold within each outer split. The same outer cross-validation folds were used across classifiers to facilitate consistent model comparisons. Stratification was used to preserve the distribution of elevated depressive symptoms across folds as closely as possible while maintaining participant-level grouping.

Within each outer training fold, hyperparameter tuning and classification-threshold selection were performed using an inner 5-fold StratifiedGroupKFold, again using SUBJNO as the grouping variable. Thus, the corresponding outer test fold remained completely independent of model preprocessing, hyperparameter selection, and threshold selection.

S4.2. Models

Three binary classification algorithms were evaluated:

- Logistic Regression
- Random Forest
- XGBoost

The same predictor set used in the primary longitudinal Model A was applied to the machine-learning analyses. Participants were not required to have complete observations across all three waves. Available observations with valid CDI outcomes were included, while missing predictor values were handled within the preprocessing pipeline as described below.

S4.3. Data Preprocessing

Data preprocessing and model training were implemented using the scikit-learn Pipeline framework.

For continuous predictors:

- Missing values were imputed using the median.
- Variables were subsequently standardized using StandardScaler.

For categorical predictors:

- Missing values were imputed using the most frequent category.
- Variables were subsequently one-hot encoded using OneHotEncoder.
- Unknown categories encountered during transformation were ignored using handle_unknown="ignore".

All preprocessing parameters were estimated exclusively from the training data within each cross-validation fold and subsequently applied to the corresponding held-out data. Preprocessing was incorporated within the model pipeline to prevent information from validation or test folds from contributing to imputation, scaling, encoding, or model fitting.

S4.4. Hyperparameter Tuning and Class Imbalance

Hyperparameters were optimized separately within each outer training fold using an inner 5-fold StratifiedGroupKFold, with participant identifiers maintained as the grouping variable. Candidate hyperparameter combinations were evaluated using average precision (AP) as the optimization metric.

For Logistic Regression, all 16 prespecified hyperparameter combinations (inverse regularization strength C, L1 or L2 penalty, and class weighting) were evaluated. For Random Forest (number of trees, maximum depth, minimum-sample constraints, maximum features, and class weighting) and XGBoost (number of estimators, maximum depth, learning rate, minimum child weight, subsampling and column-sampling ratios, regularization parameters, and positive-class weight [scale_pos_weight]), randomized search was performed with 30 candidate hyperparameter combinations evaluated for each model. Hyperparameter tuning was implemented using RandomizedSearchCV, with the number of iterations set to the full grid size for Logistic Regression.

Class imbalance was addressed as part of model development. Class-weighting parameters (class_weight for Logistic Regression and Random Forest; scale_pos_weight for XGBoost) were included among the hyperparameters considered during tuning, so that the treatment of class imbalance was selected within the inner cross-validation rather than fixed a priori. After the optimal hyperparameters were identified within an outer training fold, the selected hyperparameter values were retained for subsequent threshold selection and final model fitting within that outer fold.

S4.5. Classification Threshold Selection and Outer-Fold Evaluation

Because the classification analysis was designed to prioritize sensitivity, classification thresholds were selected using a minimum-recall criterion (recall ≥ 0.75) that was fixed before any evaluation of the outer test folds.

After hyperparameter selection within each outer training fold, out-of-fold predicted probabilities were generated using the inner 5-fold StratifiedGroupKFold with the selected hyperparameters. These inner out-of-fold predictions were used exclusively to determine the classification threshold, without reference to the corresponding outer test fold.

Among thresholds achieving a recall of at least 0.75, the threshold yielding the highest precision was selected. In the event of a tie, the largest threshold was selected. If no evaluated threshold satisfied the prespecified recall criterion, the default threshold of 0.50 was used.

The model was then refitted on the complete outer training fold using the selected hyperparameters. Predicted probabilities were generated for the corresponding held-out outer test fold, and the threshold selected exclusively from the outer training data was applied without further adjustment.

Model performance was evaluated in each outer test fold using accuracy, ROC-AUC, average precision (AP), precision, recall, specificity, false-positive rate, negative predictive value, and F1-score. Confusion-matrix counts were also recorded. Performance estimates were summarized across the 50 outer test-fold evaluations generated by the repeated 5-fold outer cross-validation.

S4.6. Model Interpretation and Subgroup Analyses

For Logistic Regression, model interpretation was based on estimated regression coefficients.

For Random Forest and XGBoost, feature-importance measures were extracted from the fitted tree-based models.

The same machine-learning procedure was repeated in:

- the overall analytic sample,
- male and female subgroups.

Supplementary Materials S5. Attrition Analysis

Attrition was evaluated by comparing baseline characteristics between participants who completed all three assessment waves and those with incomplete follow-up. Participants who did not complete all three assessment waves (n = 201) were classified as having incomplete follow-up; of these, 12 were missing the 12-month assessment only, 91 the 24-month assessment only, and 98 both follow-up assessments. Baseline demographic, gaming-related, and psychosocial characteristics were compared between the two groups, with the results presented in Table S2.

Table S2. Comparison of Baseline Characteristics between Participants Who Completed All Three Waves and Those with Incomplete Follow-Up

Variable	Overall (N=2294)	Completed all 3 waves (N=2093)	Incomplete follow-up (N=201)	SMD	P value
Demographic characteristics					
Sex, n (%)					0.4576
∟ Boy	1307 (57.0)	1187 (56.7)	120 (59.7)		
∟ Girl	987 (43.0)	906 (43.3)	81 (40.3)		
Age (years), mean ± SD	12.86 ± 1.21	12.88 ± 1.19	12.70 ± 1.38	0.145	0.0829
Socioeconomic characteristics					
Parental education level (ELPR)*, n (%)					0.0816
∟ Low	773 (33.8)	717 (34.4)	56 (28.0)		
∟ High	1513 (66.2)	1369 (65.6)	144 (72.0)		
Parental occupation status (JOBPR), n (%)					0.7816
∟ Employed	1504 (66.0)	1375 (66.1)	129 (64.8)		
∟ Unemployed / Other	776 (34.0)	706 (33.9)	70 (35.2)		
Parental marital status (MAST), n (%)					0.3500
∟ Two-parent family	2121 (92.5)	1939 (92.6)	182 (90.5)		
∟ Other (single/divorced/widowed)	173 (7.5)	154 (7.4)	19 (9.5)		
Parental living standard (LIFELV)*, n (%)					0.0741
∟ Low	411 (18.6)	374 (18.6)	37 (19.2)		
∟ Middle	1117 (50.7)	1033 (51.4)	84 (43.5)		
∟ High	676 (30.7)	604 (30.0)	72 (37.3)		
Baseline mental health					
Baseline major depressive disorder (MDD), n (%)					0.6282

Variable	Overall (N=2294)	Completed all 3 waves (N=2093)	Incomplete follow-up (N=201)	SMD	P value
∟ Yes	3 (0.1)	2 (0.1)	1 (0.5)		
∟ No	2290 (99.9)	2090 (99.9)	200 (99.5)		
PHQ-9 score, mean ± SD	2.41 ± 3.16	2.38 ± 3.11	2.71 ± 3.64	0.103	0.2232
CDI score, mean ± SD	8.24 ± 7.09	8.12 ± 6.95	9.49 ± 8.32	0.194	0.0246
Psychosocial factors					
School Bullying score (SCHOST), mean ± SD	13.34 ± 2.67	13.29 ± 2.56	13.86 ± 3.57	0.215	0.0280
Self Esteem Scale score (SES), mean ± SD	31.09 ± 5.52	31.12 ± 5.52	30.69 ± 5.45	0.078	0.2848
Social Support Appraisals Scale score (SSAS), mean ± SD	96.85 ± 16.25	97.05 ± 16.14	94.68 ± 17.25	0.146	0.0624
GMEP total score, mean ± SD	4.52 ± 1.89	4.53 ± 1.88	4.37 ± 2.01	0.089	0.2589
Gaming and media use					
Night-time gaming use, n (%)					0.1812
∟ No	1091 (56.6)	1015 (57.0)	76 (51.0)		
∟ Yes	838 (43.4)	765 (43.0)	73 (49.0)		
Weekly game time (minutes), mean ± SD	615.82 ± 693.09	610.95 ± 686.90	667.19 ± 755.29	0.081	0.3165
Log-transformed game time, mean ± SD	5.06 ± 2.59	5.03 ± 2.61	5.30 ± 2.43	0.103	0.1448
IGUESS score, mean ± SD	3.46 ± 3.86	3.39 ± 3.80	4.15 ± 4.39	0.197	0.0190
Age at first use, mean ± SD	3.53 ± 2.02	3.54 ± 2.03	3.43 ± 1.89	0.052	0.4551

Data are presented as n (%) for categorical variables and mean ± SD for continuous variables.
† Percentages are based on participants with valid responses.
* Parental education level (ELPR): High indicates college entrance or higher, whereas Low indicates high school graduation or lower.
* Parental living standard (LIFELV): Parents rated their household living standard as low, middle, or high without predefined objective criteria.
Abbreviations: CDI, Children's Depression Inventory; ELPR, parental education level; GMEP, Game Motivation and Engagement Problems; IGUESS, Internet Game Use-Elicited Symptom Screen; JOBPR, parental occupation status; LIFELV, parental living standard; MAST, parental marital status; MDD, major depressive disorder; PHQ-9, Patient Health Questionnaire-9; SD, standard deviation; SSAS, Social Support Appraisals Scale.

Supplementary Materials S6. False-Positive Burden Based on Outer-Fold Predictions

To provide a clinically interpretable assessment of the classification results and the potential false-positive burden, the numbers of true-positive (TP) and false-positive (FP) classifications per 1,000 observations were calculated from the held-out outer-fold predictions. Because the classification analyses were conducted at the observation level using repeated measurements, these quantities are reported per 1,000 observations rather than per 1,000 participants. Values were summarized across the 50 outer test-fold evaluations from the repeated nested cross-validation procedure (5 folds × 10 repetitions). To account for the clustering of repeated observations within participants, 95% confidence intervals (CIs) were estimated using 1,000 participant-level cluster bootstrap resamples of the held-out outer-fold predictions. The results are presented in Table S3.

Table S3. True-positive and false-positive classifications per 1,000 observations based on outer-fold predictions

Analysis Group	Model	TP per 1,000 Observations, Mean ± SD [95% CI]	FP per 1,000 Observations, Mean ± SD [95% CI]
Overall	Logistic Regression	41.4 ± 4.1 [35.6, 47.9]	147.4 ± 17.1 [135.0, 159.6]
	Random Forest	41.5 ± 4.1 [35.3, 47.4]	168.8 ± 22.9 [156.7, 182.1]
	XGBoost	42.0 ± 4.0 [35.9, 49.6]	163.6 ± 21.6 [150.6, 176.3]
Male	Logistic Regression	31.5 ± 4.2 [24.1, 39.2]	130.6 ± 25.3 [115.1, 146.9]
	Random Forest	31.6 ± 4.4 [24.7, 38.8]	131.9 ± 24.5 [115.9, 148.7]
	XGBoost	31.3 ± 4.7 [24.2, 38.8]	147.8 ± 31.7 [132.5, 163.1]
Female	Logistic Regression	54.2 ± 6.9 [43.4, 65.9]	183.3 ± 28.6 [162.9, 205.6]
	Random Forest	53.9 ± 6.5 [43.8, 65.9]	215.4 ± 36.9 [194.2, 237.2]
	XGBoost	54.9 ± 7.1 [43.6, 65.8]	217.6 ± 41.7 [196.2, 239.3]

Values are presented as mean ± standard deviation (SD) across the 50 outer test-fold evaluations (5 folds × 10 repetitions), with participant-level cluster bootstrap 95% confidence intervals shown in brackets. Confidence intervals were estimated using 1,000 participant-level cluster bootstrap resamples of the held-out outer-fold predictions. TP, true positive; FP, false positive; CI, confidence interval.

Across all three classifiers, the number of false-positive classifications substantially exceeded the number of true-positive classifications at the sensitivity-prioritized thresholds. In the overall sample, the models yielded approximately 41.4–42.0 true-positive classifications per 1,000 observations, compared with 147.4–168.8 false-positive classifications per 1,000 observations. A similar pattern was observed in the sex-stratified analyses. Among males, TP classifications ranged from 31.3 to 31.6 per 1,000 observations, whereas FP classifications ranged from 130.6 to 147.8. Among females, TP classifications ranged from 53.9 to 54.9 per 1,000 observations, whereas FP classifications ranged from 183.3 to 217.6. These findings demonstrate the false-positive burden associated with the sensitivity-prioritized classification thresholds and support cautious interpretation of the models for potential screening applications.
